# Exosome-mediated delivery of RBP-J decoy oligodeoxynucleotides ameliorates hepatic fibrosis in mice

**DOI:** 10.7150/thno.69885

**Published:** 2022-01-24

**Authors:** Fei He, Wei-Na Li, Xin-Xin Li, Kang-Yi Yue, Juan-Li Duan, Bai Ruan, Jing-Jing Liu, Ping Song, Zhen-Sheng Yue, Kai-Shan Tao, Lin Wang

**Affiliations:** 1 Department of Hepatobiliary Surgery, Xi-Jing Hospital, Fourth Military Medical University, Xi'an 710032, China.; 2 School of Basic Medicine, Fourth Military Medical University, Xi'an 710032, China.; 3 Xi'an Key Laboratory of Stem Cell and Regenerative Medicine, Institute of Medical Research, Northwestern Polytechnical University, Xi'an 710072, China.; 4 State Key Laboratory of Cancer Biology, Department of Biochemistry and Molecular Biology, Fourth Military Medical University, Xi'an 710032, China.

**Keywords:** Notch signaling, RBP-J, exosomes, macrophage, hepatic fibrosis

## Abstract

**Rationale:** Macrophages play multi-dimensional roles in hepatic fibrosis. Studies have implicated Notch signaling mediated by the transcription factor RBP-J in macrophage activation and plasticity. Additionally, we have previously shown that myeloid-specific disruption of RBP-J can ameliorate hepatic fibrosis in mice. Accordingly, we next asked whether blocking Notch signaling in macrophages could serve as a therapeutic strategy to treat hepatic fibrosis. In this study, we used a combination of transcription factor decoy oligodeoxynucleotides (ODNs) and exosomes to test this possibility.

**Methods:** Hairpin-type decoy oligodeoxynucleotides (ODNs) were designed for the transcription factor RBP-J. The effects of RBP-J decoy ODNs on Notch signaling were evaluated by western blot, quantitative RT-PCR, luciferase reporter assays, and electrophoretic mobility shift assays. ODNs were loaded into HEK293T-derived exosomes by electroporation. A hepatic fibrosis mouse model was established by the intraperitoneal injection of carbon tetrachloride or bile duct ligation. Mice with hepatic fibrosis were administered exosomes loaded with RBP-J decoy ODNs *via* tail vein injection. The *in vivo* distribution of exosomes was analyzed by fluorescence labeling and imaging. Liver histology was examined using hematoxylin and eosin, Sirius red, and Masson staining, as well as immunohistochemical staining for Col1α1 and αSMA.

**Results:** We found that RBP-J decoy ODNs could be efficiently loaded into exosomes and inhibit the activation of Notch signaling. Furthermore, exosomes administered *via* the tail vein were found to be primarily taken up by hepatic macrophages in mice with liver fibrosis. Importantly, RBP-J decoy ODNs delivered by exosomes could efficiently inhibit Notch signaling in macrophages and ameliorate hepatic fibrosis in mice.

**Conclusions:** Combined, our data showed that the infusion of exosomes loaded with RBP-J decoy ODNs represents a promising therapeutic strategy for the treatment of hepatic fibrosis.

## Introduction

Cirrhosis is a serious health-threatening disease with global prevalence 1. Hepatic fibrosis, a potentially reversible condition, underlies the development of cirrhosis in most chronic liver diseases 2,3. Recent reports have shown that macrophages play a central role in the pathogenesis of hepatic fibrosis and represent one of the most promising therapeutic targets for the treatment of this disease 4-6. However, macrophages cannot be simply eliminated to treat hepatic fibrosis. Mouse models of liver fibrosis have revealed that macrophages have opposing functions during the progression and regression of fibrosis 7. Accordingly, to treat this condition, it may be more meaningful to attempt to regulate the functional status of hepatic macrophages.

Notch signaling mediated by RBP-J, a transcription factor transactivated by signaling from four Notch receptors in mammals 8, has been implicated in macrophage activation and plasticity 9-11. We have previously shown that myeloid-specific disruption of RBP-J in the mouse resulted in attenuated CCl_4_- and BDL-induced liver inflammation and fibrosis 12. Based on this finding, we next proposed to treat hepatic fibrosis by inhibiting Notch signaling in macrophages. This requires that two problems be addressed, namely, how to specifically target hepatic macrophages and which Notch signaling inhibitor to use.

Exosomes are phospholipid bilayer-enclosed vesicles, 40 to 150 nm in diameter, that are secreted by cells 13,14. Owing to their excellent biocompatibility and high delivery efficiency, exosomes have enormous potential as a means for targeted drug delivery 13. In the liver, the major site for the clearance of circulating exogenous exosomes, these exosomes are predominately taken up by hepatic macrophages 15,16, underlying their potential as natural delivery systems targeting hepatic macrophages.

Transcription factor decoy (TFD) oligodeoxynucleotides (ODNs) are short, double-stranded DNA molecules distinct from antisense and siRNA oligonucleotides 17. Decoy ODNs harboring the consensus DNA recognition motif of a target transcription factor compete with the binding sites in the promoter regions of target genes for the binding of that transcription factor, thereby also significantly reducing the expression levels of downstream genes. Several studies have investigated the application of NF-κB decoy ODNs in the treatment of inflammatory disorders 18,19, highlighting the feasibility of employing RBP-J decoy ODNs for the treatment of diseases resulting from the aberrant activation of Notch signaling.

In this study, we found that RBP-J decoy ODNs delivered *via* exosomes could efficiently inhibit Notch signaling in macrophages and ameliorate liver fibrosis in mice.

## Materials and Methods

### Synthesis and annealing of decoy ODNs

The RBP-J decoy was a single-stranded ODNs that could form a stem-loop structure after annealing. Two RBP-J binding sites (CGTGGGAA 20) were present in the decoy, with three phosphorothioate-modified sites at each end. For the control decoy, a mutation was introduced in the RBP-J binding sites within the ODNs. The ODNs were labeled either with biotin or FAM at the 5′ end in some experiments. All the ODNs were synthesized by AuGCT Dingsheng Biotechnology Co. (Beijing, China). The decoy ODNs were resuspended at a concentration of 20 μM, incubated for 10 min at 95 °C, and then slowly cooled to room temperature. The sequence of the RBP-J decoy ODNs (Decoy RBP-J) was 5′-CTGCGTGGGAACTAGCGTGGGAATATTTTTTATATTCCCACGCTAGTTCCCACGCAG-3′; the sequence of the control decoy ODNs (Decoy Ctrl) was 5′-CTGCGTTTTAACTAGCGTTTTAATATTTTTTATATTAAAACGCTAGTTAAAACGCAG-3′.

### Cell culture and transfection

Bone marrow-derived macrophages (BMDMs) were cultured as previously described 9. In some cases, lipopolysaccharide (LPS, 200 ng/mL; Sigma‐Aldrich, St. Louis, MO, USA) was included in the medium. Hepatic macrophages were isolated using magnetic-activated cell sorting (MACS) as previously described 21. HEK293T cells and mouse RAW264.7 macrophages were cultured in Dulbecco's modified Eagle's medium (DMEM) supplemented with 10% fetal bovine serum (FBS), 2 mM L-glutamine, 100 U/mL penicillin, and 100 μg/mL streptomycin in a humidified atmosphere with 5% CO_2_ at 37 °C. Human umbilical vein endothelial cells (HUVECs) were cultured in endothelial cell medium (Sciencell, San Diego, CA, USA) supplemented with 5% FBS, 1% endothelial cell growth supplements (Sciencell), and antibiotics.

RAW264.7 macrophages or HUVECs were transfected with RBP-J decoy or control decoy ODNs (100 pmol) using HiPerFect Transfection Reagent (QIAGEN, Crawley, UK) according to the manufacturer's instructions. After 24 h, transfected RAW264.7 cells were infected with Ad-NICD (Notch intracellular domain) adenovirus (HanBio, Shanghai, China) while transfected HUVECs were placed in Dll4-Fc (1 μg/mL; Sino Biological, Beijing, China)-coated culture plates after digestion.

### Luciferase reporter assay

HeLa cells (2 × 10^4^) were transfected with 100 ng of pGa9816, 50 ng of pEFBOS-NICD, the indicated amounts of Decoy RBP-J or Decoy Ctrl ODNs, and 5 ng of pRL-TK (internal control) using Lipofectamine 2000 (Invitrogen, Carlsbad, CA, USA) according to the protocol recommended by the manufacturer. Cells were collected 24 h after transfection. Luciferase activity was assessed using a Dual-Luciferase Reporter Assay Kit (Promega, Madison, WI, USA) and assay results were read using a Luminoskan Ascent Luminescence Plate Reader (Labsystems, Helsinki, Finland).

### Electrophoretic mobility shift assay (EMSA)

Binding between Decoy RBP-J ODNs and RBP-J was detected using an EMSA kit (Beyotime, Shanghai, China) following the manufacturer's protocol. HEK293T cells were transfected with pCMV-RBPJ-Flag and lysed in cell lysis buffer (Beyotime). The protein concentration was determined using a BCA Protein Assay Kit (Pierce, Rock, IL, USA). Cell extracts (20 μg) were incubated with biotin-labeled RBP-J decoy ODNs (3.5 μM, 1 μL) in EMSA/Gel Shift binding buffer for 20 min at room temperature. In competition experiments, 1 μL of unlabeled RBP-J decoy ODNs (35 μM) was added during the DNA-protein incubation period. In non-specific competition experiments, unlabeled control decoy ODNs (35 μM, 1 μL) were used. For antibody supershift studies, an anti-Flag antibody (Cell Signaling Technology, Danvers, MA, USA) was used. The DNA-protein complexes were separated using 6% native-polyacrylamide gel electrophoresis (PAGE) at 70 V for 1 h and then transferred to a positively charged nylon membrane (Beyotime) at 380 mA for 1 h. The membrane was subsequently cross-linked using UV light, incubated with horseradish peroxidase (HRP)-conjugated streptavidin, and detected using chemiluminescence reagents (BeyoECL Moon; Beyotime).

### RNA extraction and quantitative reverse transcription PCR (qRT-PCR)

Total RNA was isolated from RAW264.7 macrophages, HUVECs, BMDMs, exosomes, or liver tissues with Trizol reagent (Invitrogen) according to the manufacturer's instructions. MiRNA and mRNA were reverse transcribed into cDNA using respectively the Mir-X miRNA qRT-PCR SYBR Kit and PrimeScript RT Master Mix (Takara, Dalian, China). qRT-PCR was performed with the TB Green Premix EX Taq II (Tli RNaseH Plus) Kit (Takara) in a Quantstudio 5 Real-Time PCR System (Bio-Rad, Hercules, CA, USA). U6 (miRNA) or β-actin (mRNA) served as the internal control. The sequences of the primers used are listed in Table S1.

### Western blot

Cells and exosomes were lysed with radioimmunoprecipitation assay (RIPA) buffer (Beyotime) containing phenylmethylsulfonyl fluoride. The protein concentration was determined using the BCA Protein Assay Kit (Pierce) following the manufacturer's instructions. The samples were analyzed by sodium dodecyl sulfate (SDS)-PAGE, transferred to polyvinylidene fluoride membranes (Millipore, Billerica, MA, USA), and incubated first with primary antibodies targeting Hes1, CD9, Alix, flotillin-1 (Cell Signaling Technology), HEY1, β-actin (Proteintech, Wuhan, China), and VDAC1 (Abcam, Cambridge, UK) and then with HRP-conjugated goat anti-mouse or anti-rabbit IgG secondary antibodies (Zhuangzhi Bio, Xi'an, China).

### Exosome isolation and labeling

HEK293T cells were cultured in FBS-free medium for 24-36 h. Exosomes in supernatants were isolated using PEG6000 (Sigma) as previously described 22. In brief, the culture medium was collected and centrifuged first at 500 × *g* for 5 min and then at 3,000 × *g* for 15 min to remove cells and cell debris. The supernatants were then filtered through a 0.22-μm filter (Millipore) and mixed with PEG6000 working solution to a final concentration of 12% PEG6000. The mixtures were incubated at 4 °C for 12 h, centrifuged at 12,000 × *g* for 60 min, and the resulting pellets were resuspended in phosphate-buffered saline (PBS). The size distribution of the exosomes was analyzed by flow cytometry for nanoparticle analysis (NanoFCM, Xiamen, China) and exosome morphology was observed by transmission electron microscopy (TEM). For labeling, exosomes in PBS were incubated with Dil (Invitrogen) at 37 °C for 30 min according to the manufacturer's protocol, mixed with PEG6000 working solution, and then centrifuged at 12,000 × *g* for 30 min.

For BMDMs, 2-8 μg (protein equivalent) of exosomes was added to 24-well plates containing BMDMs followed by 24-48 h of culture. In some experiments, BMDMs were fixed for 15 min in 4% paraformaldehyde and the nuclei were counterstained with Hoechst 33258 (Sigma). The fluorescence signals of the labeled exosomes and nuclei were imaged using a fluorescence microscope (BX51, Olympus, Tokyo, Japan).

### ODNs loading

Exosomes were electroporated with decoy ODNs, FAM-ODNs, or miR-140-5p (RiboBio, Guangzhou, China) at 400 V and 125 μF in 0.4-cm electroporation cuvettes as previously described 23. For cell culture, 2-8 μg (protein equivalent) of exosomes were electroporated with 5 μL of nucleotides (20 μM); for administration to mice, 200 μg of exosomes were electroporated with 2.5 nmol of ODNs. To remove free ODNs, exosomes were washed and centrifuged with PBS containing 12% PEG6000. To determine the efficiency of electroporation, the level of miR-140-5p was detected by qRT-PCR. In brief, a serial dilution (10 ×) of miR-140-5p mimics (20 pmol) was used to generate a standard curve of Ct values and mimic contents through qRT-PCR. Then, the Ct value for miR-140-5p in exosomes was measured by qRT-PCR and converted to the miR-140-5p content in exosomes. The percentage of miR-140-5p entering exosomes was calculated based on the total amount of miR-140-5p mimics added before electroporation.

### Mouse model of hepatic fibrosis

To induce hepatic fibrosis, male C57BL/6 mice were intraperitoneally injected with 0.75 mL/kg CCl_4_ (Tianli, Tianjin, China) diluted in sterile olive oil twice a week for 6 weeks, with olive oil only serving as control. Three days after the last injection, mice were euthanized for further experiments. In some experiments, hepatic fibrosis was also induced by extrahepatic cholestasis resulting from bile duct ligation (BDL). Mice were analyzed 2 weeks after the operation. All animal experiments were performed following the guidelines of the Animal Experiment Administration Committee of the university.

### Exosome injection and histological analysis

For *in vivo* exosome tracking, fibrotic mice were injected with Dil-labeled exosomes *via* the tail vein. Tissues were harvested 6 and 48 h after injection for bioluminescence imaging (Andor iKon Dw434, Oxford Instruments, Oxon, UK) or tissue sectioning.

For exosome-mediated therapy, exosomes derived from HEK293T cells were loaded with RBP-J decoy or control decoy ODNs and infused into mice with CCl_4_-induced fibrosis (on days 29, 32, 36, and 39) or those with BDL-induced fibrosis (on days 2, 5, 8, and 11) four times by tail vein injection. Mice were humanely euthanized for further analysis 2-3 days after the last exosome injection.

For immunofluorescence staining, liver tissues were fixed in 4% paraformaldehyde for 15 min and stained first with an anti-mouse F4/80 antibody (eBioscience, San Diego, CA, USA) and then with Alexa Fluor 488-labeled donkey anti-rat IgG (Invitrogen). Nuclei were counterstained with Hoechst 33258 (Sigma).

Formaldehyde-fixed liver tissues were paraffin-embedded, sliced into 6-μm-thick sections, and subjected to hematoxylin and eosin (H&E), Sirius red, and Masson staining according to standard protocols. For immunohistochemistry, sections from mouse livers were prepared according to standard procedures. The primary antibodies used were anti-mouse Col1α1 (Servicebio, Wuhan, China) and anti-mouse αSMA (Servicebio). HRP-conjugated goat anti-rabbit IgG was used as the secondary antibody. 3,3′-Diaminobenzidine (DAB) was used as the chromogen (Servicebio). Images were captured using a digital slide scanner (Pannoramic 250/midi, 3DHISTECH, Hungary).

### Biochemistry

Serum albumin, alanine aminotransferase (ALT), and aspartate aminotransferase (AST) levels were determined using an automatic biochemical analyzer (Rayto Life and Analytical Sciences Company, Shenzhen, China).

### Enzyme-linked immunosorbent assay (ELISA)

The levels of interleukin-1 beta (IL-1β) and IL-6 in culture supernatant were measured using kits from Thermo Fisher Scientific (MA, USA) following the manufacturer's protocols.

### Statistical analysis

Images were imported into Image-Pro Plus 6.0 (Media Cybernetics Inc., Bethesda, MD, USA). Data were analyzed with GraphPad Prism software, version 8.0. Comparisons between groups were undertaken using paired or unpaired Student's *t*-tests. Non-normally distributed data were analyzed using the Mann-Whitney test. The number of repetitions is indicated in the legends of each graph. Results were expressed as means ± SD. *P*-values <0.05 were considered significant.

## Results

### RBP-J decoy ODNs inhibited Notch signaling activation

**Figure 1:**
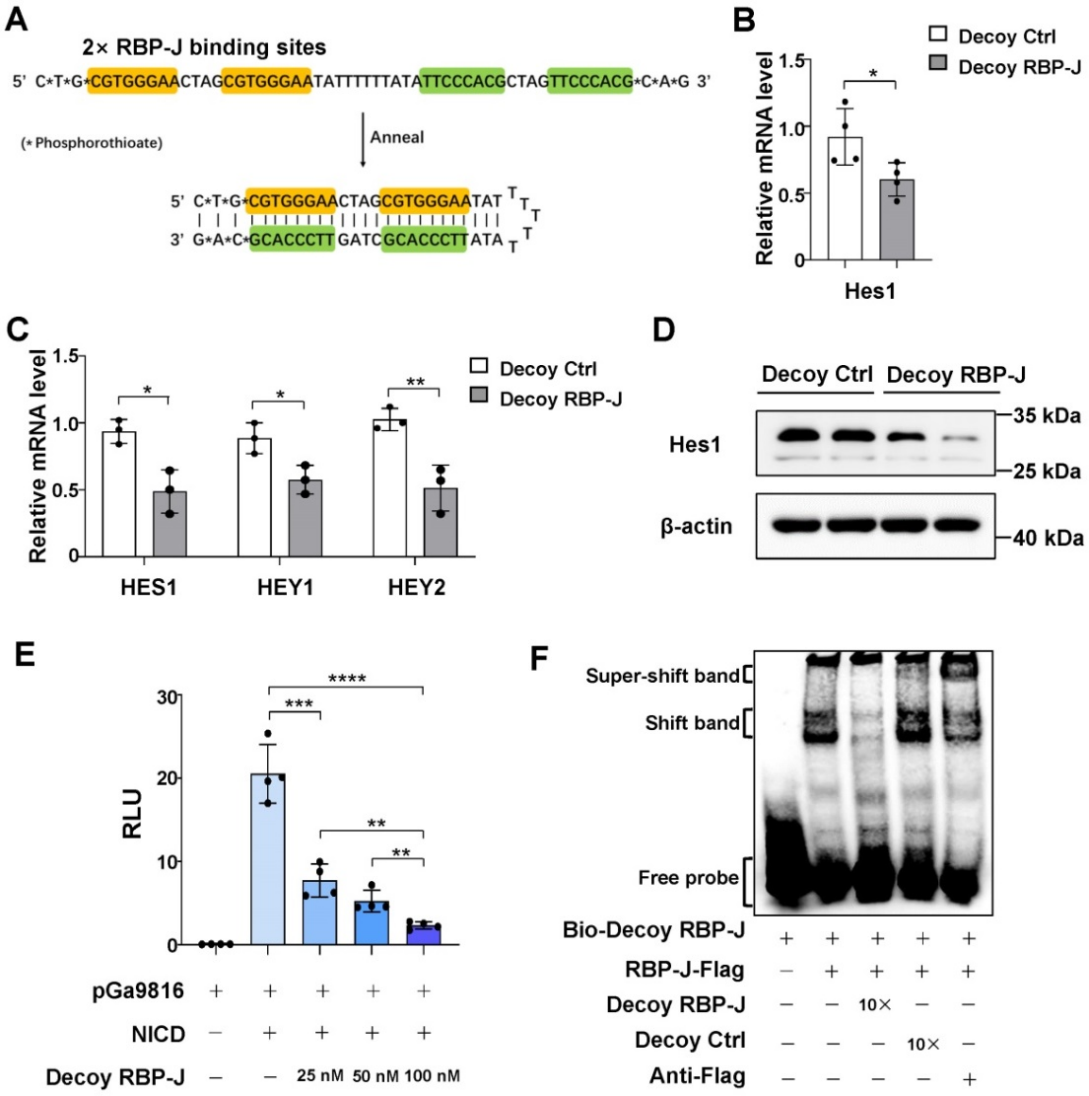
** RBP-J decoy oligodeoxynucleotides (ODNs) inhibited Notch signaling activation.** (A) The structure of the decoy ODNs for the transcription factor RBP-J. (B-D) RAW264.7 cells or HUVECs were transfected with RBP-J decoy ODNs (Decoy RBP-J) or control decoy ODNs (Decoy Ctrl). After 24 h, Notch signaling was activated by the overexpression of the Notch intracellular domain (NICD) (Ad-NICD) in RAW264.7 cells or with Dll4-Fc in HUVEC cells. The mRNA levels of Notch target genes were detected by qRT-PCR in RAW264.7 cells (B) or HUVECs (C). The expression of Hes1 in RAW264.7 cells was determined by western blot, with β-actin serving as a reference control (D). (E) Luciferase reporter assay. HeLa cells were co-transfected with the RBP-J reporter plasmid pGa9816, pEFBOS-NICD, and increasing concentrations of RBP-J decoy ODNs; pRL-TK served as a reference control. Luciferase activity was detected 24 h after transfection. (F) Representative electrophoretic mobility shift assay (EMSA) results showing the binding of RBP-J to RBP-J decoy ODNs. HeLa cells were transfected with pCMV-RBP-J-Flag. Next, cell extracts were incubated in EMSA/Gel Shift binding buffer in the presence of biotin-labeled RBP-J decoy ODNs, unlabeled RBP-J decoy ODNs, control decoy ODNs, or an anti-Flag antibody, as indicated. Bars = means ± SD; * *P* < 0.05, ** *P* < 0.01, *** *P* < 0.001, **** *P* < 0.0001.

We designed a hairpin-type decoy oligodeoxynucleotide targeting the transcription factor RBP-J. As shown in Figure 1A, the Decoy RBP-J ODNs was a single-stranded ODN that can form a hairpin-type structure after annealing. Two RBP-J binding sites were present in the Decoy RBP-J ODNs as well as three phosphorothioate-modified sites at each end to enhance the stability of the ODNs 17.

Next, we evaluated whether RBP-J decoy ODNs could block Notch signaling from five aspects. First, we measured the mRNA levels of target genes downstream of Notch signaling in RAW264.7 macrophages and HUVECs transfected with RBP-J decoy ODNs. qRT-PCR analysis showed that RBP-J decoy ODNs induced a significant reduction in the mRNA levels of hairy and enhancer of split 1 (*Hes1*) in RAW264.7 cells (Figure 1B) and those of *HES1*, Hes-related family bHLH transcription factor with YRPW motif 1 (*HEY1*), and *HEY2* in HUVEC's compared with that in cells treated with control decoy ODNs (Figure 1C). Second, we assessed the protein levels of Notch target genes in RAW264.7 macrophages and HUVECs. Compared with controls, western blot results demonstrated that the expression of HES1 and/or HEY1 was decreased in RBP-J decoy ODNs-treated RAW264.7 cells and HUVECs (Figure 1D, Figure S1A). Third, we performed a luciferase reporter assay in HeLa cells using the pGa9816 plasmid, the promoter region of which contains several RBP-J binding sites 24. We found that luciferase expression was activated following transfection with the NICD; however, co-transfection with RBP-J decoy ODNs reduced luciferase activity, an effect that was found to be concentration-dependent (Figure 1E). Fourth, we performed an EMSA to confirm the direct binding between RBP-J decoy ODNs and RBP-J (Figure 1F). We found that RBP-J could bind to biotin-labeled RBP-J decoy ODNs, as indicated by the appearance of a shifted band; however, this binding was abrogated by competition with a 10-fold molar excess of unlabeled RBP-J decoy ODNs, but not with the same concentration of mutated ODNs (Decoy Ctrl). Moreover, a supershifted band was generated in the presence of an anti-RBP-J antibody. Finally, we determined whether RBP-J decoy ODNs could prevent the binding of RBP-J to the *HEY1* promoter region using a chromatin immunoprecipitation (ChIP) assay. For this, HeLa cells were co-transfected with the pCMV-RBPJ-Flag vector and RBP-J decoy ODNs. ChIP was performed using an anti-Flag, rabbit IgG, or anti-histone H3 antibody. Human *HEY1* promoter fragments (-180 to -65) encompassing one RBP-J binding site in both input and precipitated DNA were detected by qRT-PCR. As shown in Figure S1B, RBP-J decoy ODNs could impair the binding of RBP-J to the *HEY1* promoter. Combined, these results suggested that RBP-J decoy ODNs could inhibit Notch signaling activation.

### Characterization of exosomes derived from HEK293T cells and the efficient loading of exosomes with ODNs using electroporation

**Figure 2:**
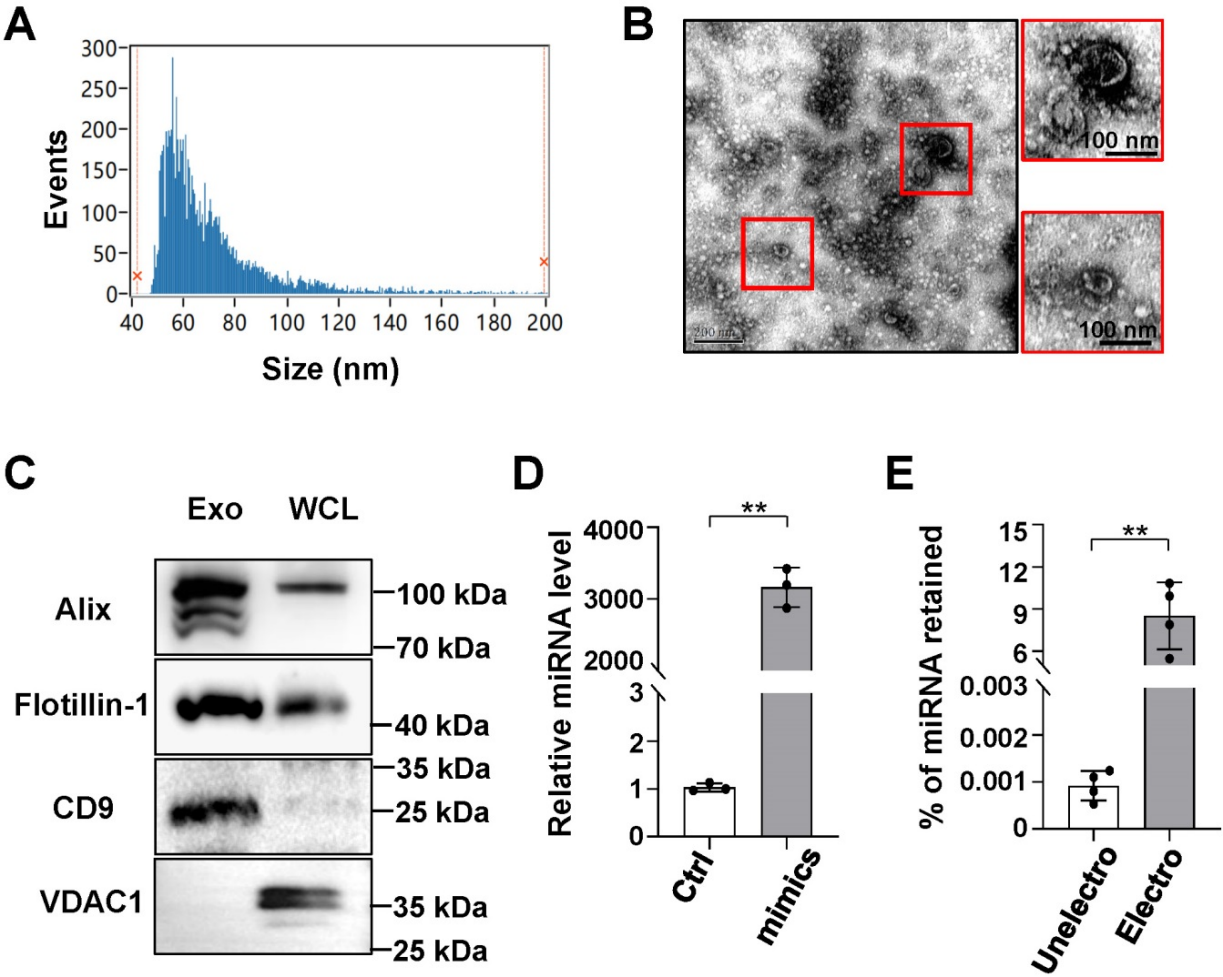
** Characterization of HEK293T-derived exosomes and retention of oligonucleotides in exosomes after electroporation.** (A) The size distribution of exosomes was determined by flow cytometry for nanoparticle analysis. (B) Exosome morphology was observed by transmission electron microscopy (TEM). (C) The levels of CD9, Alix, flotillin-1, and VDAC1 in exosomes of HEK293T lysates were determined by western blot. (D) Oligonucleotides (miR-140-5p) were detected in exosomes that had been electroporated with scrambled miRNAs (Ctrl) or miR-140-5p mimics using qRT-PCR; U6 served as a reference control. (E) The percentage of oligonucleotides (miR-140-5p) retained in exosomes before or after electroporation was calculated according to qRT-PCR standard curves. Bars = means ± SD; ** *P* < 0.01.

Although transcription factor decoy-based strategies have shown biomedical potential, the safe and efficient delivery of decoy ODNs remains a barrier to their broader clinical application 18. Furthermore, exosomes have shown potential as a novel method for drug delivery owing to their high delivery efficiency and biocompatibility 13,14. Accordingly, we sought to determine the feasibility of delivering decoy ODNs using exosomes isolated from culture supernatants of HEK293T cells. Particle size analysis showed that HEK293T-derived exosomes were approximately 63.75 ± 22.28 nm (median ± SD) in diameter (Figure 2A). TEM indicated that the exosomes had a bilayer structure (Figure 2B). Western blot analysis revealed an enrichment of exosomal marker proteins, including Alix, flotillin-1, and CD9 25, whereas the expression of mitochondrial membrane protein VDAC1 was barely detectable (Figure 2C).

Next, ODNs were loaded into exosomes by electroporation, as previously reported 23. To quantify ODNs in exosomes, miR-140-5p mimics were utilized for convenience of detection when using qRT-PCR. Exosomes (5 μg protein equivalent) were electroporated with miR-140-5p mimics or negative control mimics (100 pmol). The qPCR results showed that the level of miR-140-5p in the loaded exosomes was approximately 3,000-fold that of the control miRNA (Figure 2D), accounting for approximately 9% of the electroporated miRNA (Figure 2E). These data confirmed that exosomes could efficiently load ODNs.

### Exosomes delivered via the tail vein were mainly taken up by hepatic macrophages in a murine model of hepatic fibrosis

**Figure 3:**
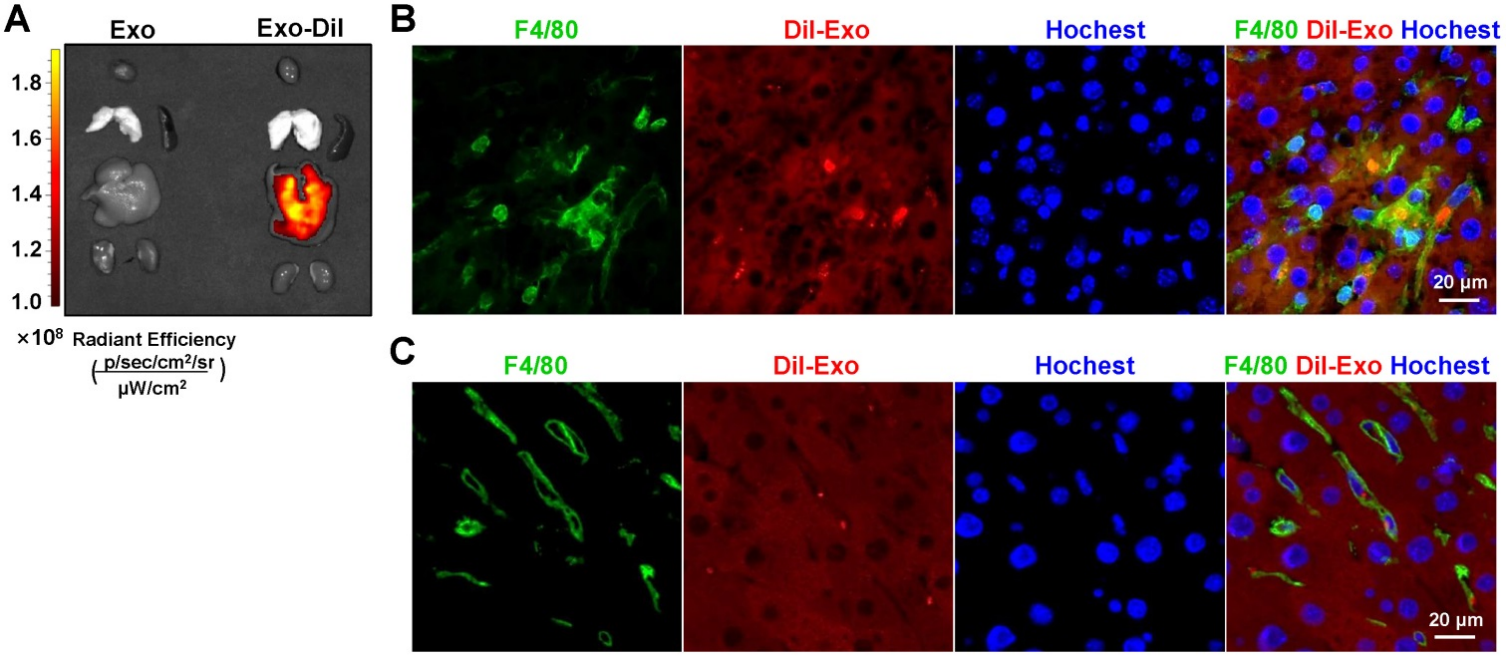
** Exosomes delivered by tail vein injection were mainly taken up by hepatic macrophages in mice with CCl_4_-induced fibrosis.** Exosomes were stained with Dil and approximately 200 µg (protein equivalent) of exosomes in 150 µL of PBS were injected into mice with CCl_4_-induced fibrosis *via* the tail vein. (A) After 6 h, Dil signals in the liver, lung, spleen, kidney, and heart were examined using bioluminescence imaging. After 6 (B) or 48 h (C) of injection of Dil-labeled exosomes, liver sections were stained with an anti-mouse F4/80 antibody and analyzed by fluorescence microscopy. Nuclei were counterstained with Hoechst 33258.

In the liver, the major site for exosome removal from the systemic circulation, exosomes are predominantly taken up by macrophages 15,16. Accordingly, we sought to determine whether exogenous exosomes would have a similar fate. To test this, we generated a mouse model of hepatic fibrosis by intraperitoneal injection of CCl_4_ (Figure 5A) or bile duct ligation (Figure S4A). Mice were infused with Dil-labeled exosomes (200 μg/mouse). As expected, bioluminescence imaging analysis revealed that Dil-labeled exosomes were mainly distributed in the liver 6 h after injection (Figure 3A). Moreover, immunofluorescence staining of liver sections showed the presence of Dil-labeled exosomes in F4/80^+^ macrophages 6 h and 48 h after injection (Figure 3B, C; Figure S2). These findings suggested that HEK293T-derived exosomes were taken up by hepatic macrophages in fibrotic mice.

### Exosomes loaded with RBP-J decoy ODNs inhibited Notch signaling activation in macrophages

**Figure 4:**
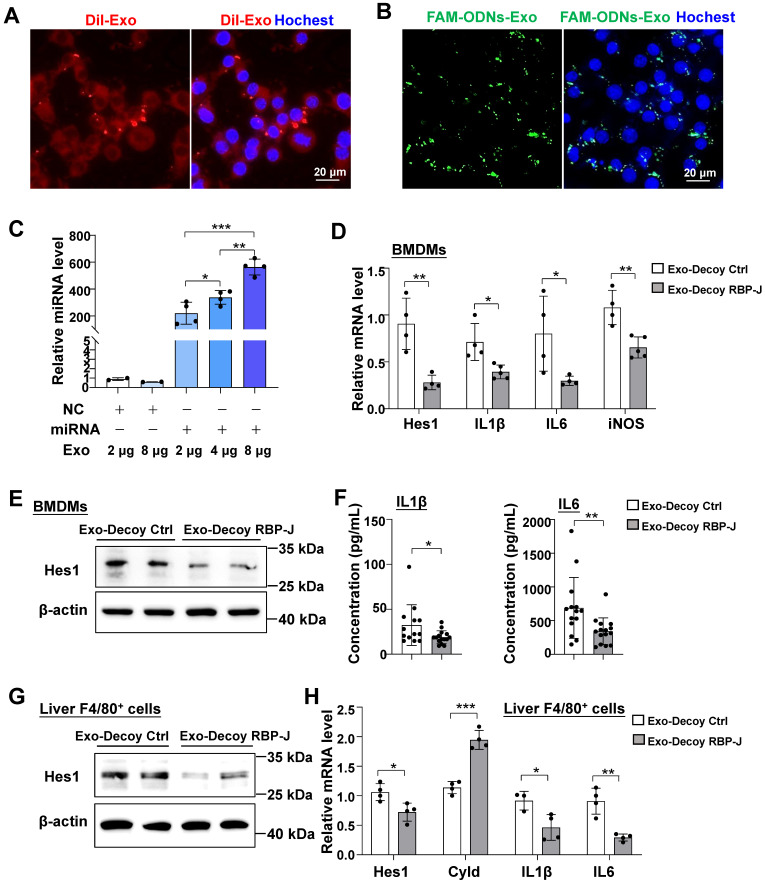
** Exosomes loaded with RBP-J decoy oligodeoxynucleotides (ODNs) inhibited Notch signaling in macrophages.** Bone marrow-derived macrophages (BMDMs) were incubated with Dil-exosomes (A) or exosomes loaded with FAM-labeled ODNs (B) for 24 h and then analyzed by fluorescence microscopy. Nuclei were counterstained with Hoechst 33258. (C) Exosomes were loaded with oligonucleotides (miR-140-5p mimics or control mimics, 100 pmol) by electroporation and different concentrations of the loaded exosomes were incubated with BMDMs. After 24 h, the level of miR-140-5p was quantified in the BMDMs by qRT-PCR, with U6 serving as a reference control. (D-F) BMDMs were incubated with exosomes that had been electroporated with RBP-J decoy or control decoy ODNs for 24 h and then stimulated with LPS (200 ng/mL) for 24 h. The mRNA levels of Hes1, IL1β, IL6, and iNOS in BMDMs were detected by qRT-PCR (D). The protein level of Hes1 was determined by western blot (E), with β-actin serving as a reference control. The levels of IL1β and IL6 in culture supernatant were detected by ELISA (F). (G, H) Mice were intraperitoneally injected with CCl_4_ twice a week for 6 weeks; exosomes loaded with RBP-J decoy or control decoy ODNs (exosomes/decoy ODNs = 200 ng/2.5 nmol) were infused into mice four times *via* tail vein injection. F4/80^+^ hepatic macrophages were isolated using magnetic-activated cell sorting (MACS). The protein level of Hes1 was determined by western blot (G) and the mRNA levels of Hes1, Cyld, IL1β, and IL6 in F4/80^+^ hepatic macrophages were measured by qRT-PCR (H). Bars = means ± SD; * *P* < 0.05, ** *P* < 0.01, *** *P* < 0.001.

In line with the Dil labeling results *in vivo*, we found that BMDMs could also phagocytose Dil-labeled exosomes after 24 h of co-culture (Figure 4A). Exosomes loaded with FAM-labeled ODNs *via* electroporation were also incubated with BMDMs for 24 h. Fluorescence imaging showed that exosomes could deliver FAM-labeled ODNs into macrophages (Figure 4B). Different concentrations of exosomes (2-8 μg protein equivalent) were loaded with equal concentrations of miR-140-5p mimics (100 pmol) *via* electroporation and then co-cultured with BMDMs. After 24 h, the level of miR-140-5p in BMDMs was detected using qRT-PCR. The results indicated that electroporation of miRNA-loaded exosomes led to a significant upregulation of miR-140-5p levels in BMDMs when compared with the control condition (Figure 4C). These data suggested that exosomes could deliver ODNs into macrophages *in vitro*.

**Figure 5:**
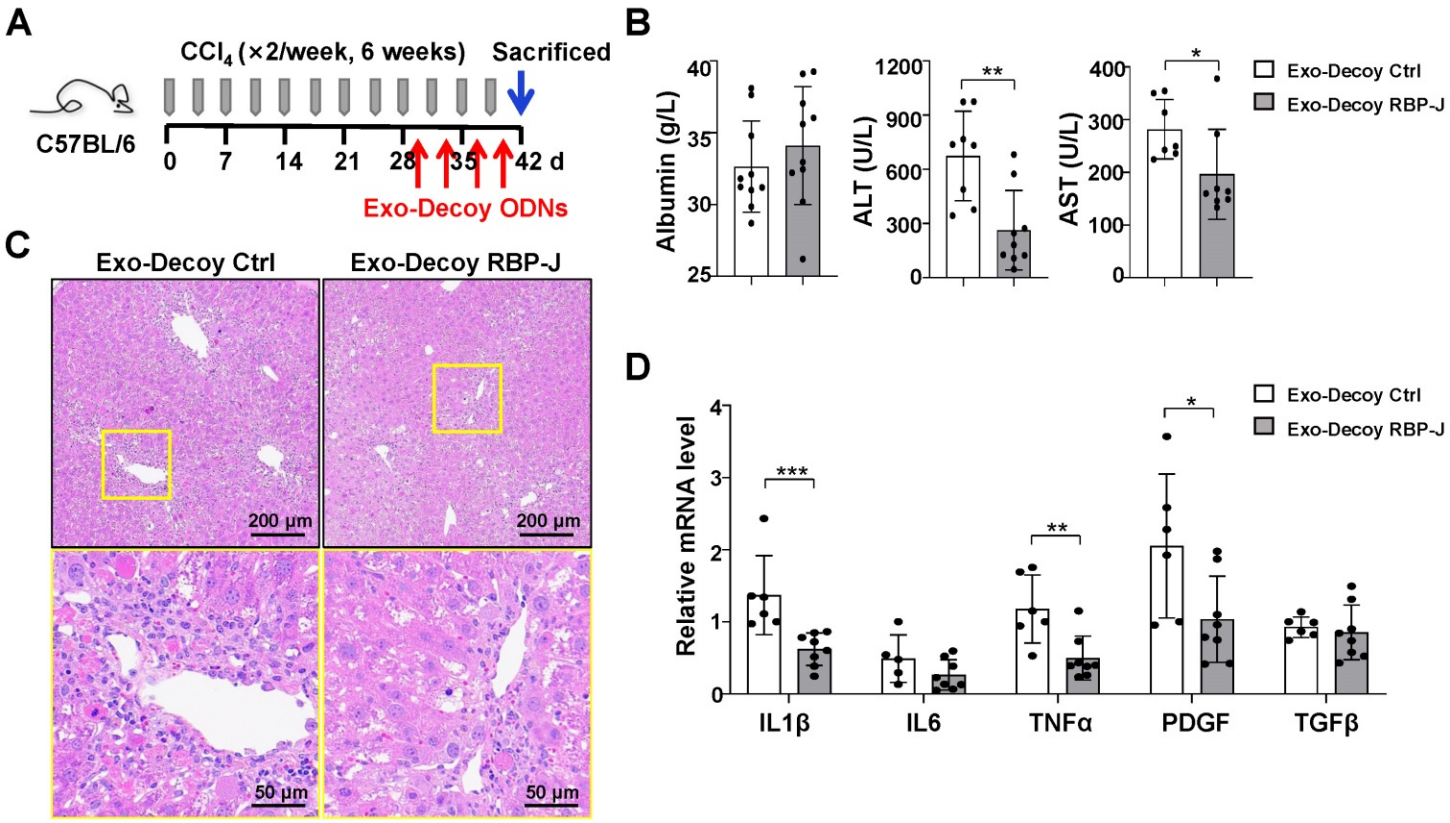
** Exosomes loaded with RBP-J decoy oligodeoxynucleotides (ODNs) attenuated liver inflammation in mice with CCl_4_-induced fibrosis.** (A) Schematic illustration of the procedure used for the loading of exosomes with RBP-J decoy ODNs to treat liver fibrosis in mice. (B) Liver sections were subjected to hematoxylin and eosin (H&E) staining. The micrographs in the lower row are a higher magnification of the yellow frames in the upper row. (C) Serum albumin, alanine aminotransferase (ALT), and aspartate aminotransferase (AST) levels in the mice. (D) The mRNA levels of IL1β, IL6, TNF-α, PDGF-B, and TGFβ in the liver were determined by qRT-PCR. Bars = means ± SD; * *P* < 0.05, ** *P* < 0.01, *** *P* < 0.001.

We next assessed if exosomes loaded with RBP-J ODNs (Exo-Decoy RBP-J ODNs) were functional in macrophages. For this, BMDMs were cultured with Exo-Decoy RBP-J ODNs for 24 h and then stimulated with LPS (200 ng/mL) for 24 h, following which the expression levels of Hes1 were evaluated. As shown in Figure 4D, E, Exo-Decoy RBP-J ODNs reduced the mRNA and protein levels of Hes1 in BMDMs. In line with our previously reported results in RBP-J-knockout BMDMs 9,12, culture with Exo-Decoy RBP-J ODNs also led to the inhibition of the expression of inflammatory factors such as IL1β, IL6, and/or inducible nitric oxide synthase (iNOS) in BMDMs (Figure 4D, F). Fibrotic mice were also injected with Exo-Decoy RBP-J or Exo-Decoy Control ODNs *via* the tail vein (Figure 5A). Hepatic F4/80^+^ cells were isolated using MACS, reaching 85% purity (Figure S3). The expression of Hes1, IL1β, and IL6 was downregulated, whereas that of Cyld, a deubiquitinase negatively correlated with Notch signaling 12, was upregulated in hepatic F4/80^+^ cells derived from Exo-Decoy RBP-J ODN-treated mice compared with that in mice treated with control ODNs (Figure 4G, H). These results suggested that Exo-Decoy RBP-J ODNs could inhibit Notch signaling in macrophages both *in vitro* and* in vivo*.

### Exo-RBP-J decoy ODNs ameliorated liver inflammation and fibrosis in mice with CCl_4_- or BDL-induced fibrosis

Next, we evaluated the efficacy of Exo-Decoy RBP-J ODNs in a mouse model of CCl_4_- or BDL-induced liver fibrosis. For CCl_4_-induced fibrosis, mice were administered CCl_4_ twice a week for 6 weeks and then infused with Exo-Decoy RBP-J or Exo-Decoy Control ODNs (200 μg/mouse) *via* the tail vein 24 h after the 9^th^ to 12^th^ injection of CCl_4_ (Figure 5A). For BDL-induced fibrosis, mice were injected with Exo-Decoy RBP-J or Exo-Decoy Control ODNs *via* the tail vein on days 2, 5, 8, and 11 (Figure S4A). The results showed that serum ALT and AST levels were decreased in Exo-Decoy RBP-J ODN recipient mice compared with that in mice administered control ODNs (Figure 5B, Figure S4B), suggesting that liver function had improved in mice treated with RBP-J Decoy ODNs. Moreover, H&E staining of liver sections showed that the portal region of livers from Exo-Decoy RBP-J ODN-treated mice had fewer inflammatory cells relative to that in the livers of control ODN-treated animals (Figure 5C, Figure S4C).

**Figure 6:**
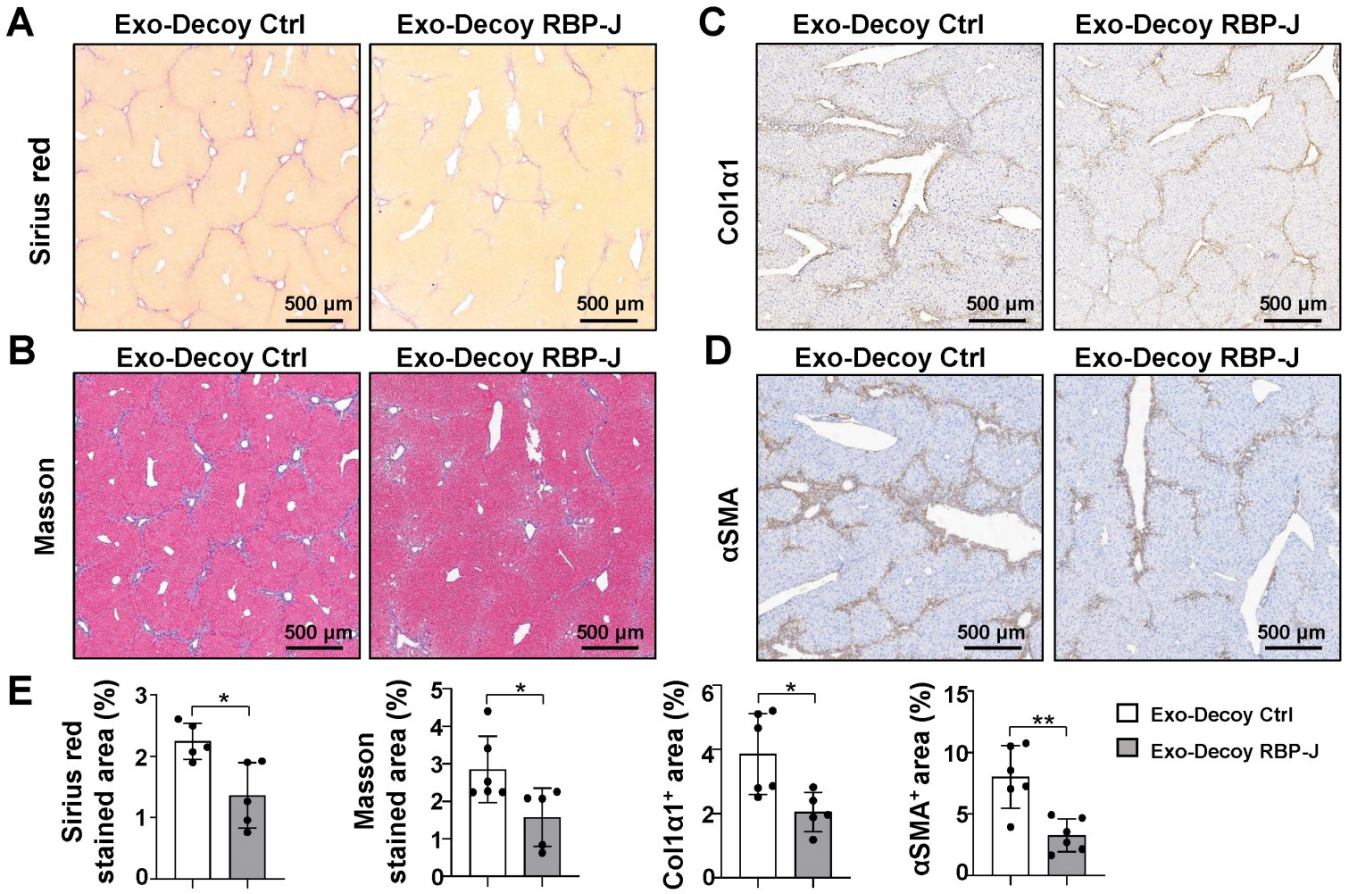
**Exosomes loaded with RBP-J decoy oligodeoxynucleotides (ODNs) ameliorated liver fibrosis induced by CCl_4_ in mice.** Liver sections were subjected to Sirius red (A) and Masson staining (B). Liver sections were subjected to immunohistochemical staining for Col1α1 (C) and αSMA (D). (E) Areas positive for Sirius red staining in (A), Masson staining in (B), Col1α1 in (C), and αSMA in (D) were quantitatively compared. Bars = means ± SD; * *P* < 0.05, ** *P* < 0.01.

The mRNA expression of inflammatory and fibrosis factors was determined using qRT-PCR. The results indicated that the production of the inflammatory factors IL1β and TNFα and the fibrosis factors PDGF-B and/or TGFβ was significantly decreased in the livers of mice infused with Exo-Decoy RBP-J ODNs (Figure 5D, Figure S4D). Sirius red, Masson, and Col1α1 staining showed that Exo-Decoy RBP-J ODNs administration significantly reduced ECM deposition compared with the administration of control ODNs (Figure 6A-C, E; Figure S5A-C, E; Figure S6A, C). Hepatic stellate cells (HSCs) are the major source of the components of the fibrous scar in liver fibrosis 2,3. Accordingly, we determined the effect of Exo-Decoy RBP-J ODNs on HSCs activation *via* staining with an anti-αSMA antibody. The results indicated that the infusion of Exo-Decoy RBP-J ODNs could reduce the αSMA-positive signals, suggesting that HSCs activation had been abrogated (Figure 6D, E; Figure S5D, E; Figure S6B, D). Overall, these data suggested that Exo-Decoy RBP-J ODNs application could attenuate liver inflammation and hepatic fibrosis in mice.

## Discussion

Notch signaling plays a role in the development of hepatic fibrosis by regulating different cell types 26. Zhu et al. 27 reported that hepatocyte-specific Notch loss-of-function resulted in the attenuation of non-alcoholic steatohepatitis (NASH)-associated liver fibrosis, while forced activation of Notch signaling in the liver could induce fibrosis. Furthermore, we have previously shown that myeloid-specific disruption of RBP-J using Lyz2-Cre RBP-J^flox/flox^ or RBP-J deficiency in hepatic myofibroblasts mediated by Sm22α-Cre^ER^ RBP-J^flox/flox^ could reduce hepatic fibrosis in mice 12,28, while endothelial Notch activation with CDH5-Cre^ER^ NICD elicited the opposite effect 29. These results suggested that blocking Notch signaling in hepatocytes, macrophages, liver sinusoidal endothelial cells, or HSCs can exert a beneficial effect on hepatic fibrosis in mice. However, the systematic blockade of Notch signaling is not a feasible therapeutic option for this condition given the extensive role of Notch in development. For instance, systemic γ-secretase inhibitor (GSI) treatment can cause goblet cell metaplasia 30. Indeed, when using a Cre-inducible transgenic mouse model (Mx-Cre), we found that the systemic knockout of RBP-J in adult mice, in which RBP-J is deleted with high efficiency in both the hematopoietic system and the liver, led to the aggravation of CCl_4_-induced hepatic fibrosis (data not shown).

The above observations highlight the need to develop therapeutic strategies that rely on tissue- or cell-specific inhibition of Notch signaling for the treatment of hepatic fibrosis. Pajvani et al. 31 developed a nanoparticle-mediated delivery system targeting GSI to the liver and demonstrated that GSI-nanoparticle application reduced hepatic fibrosis and inflammation in mice. In addition, macrophages are known to play a central role in the pathogenesis of liver fibrosis and we have previously shown that myeloid-specific blockade of Notch signaling ameliorated hepatic fibrosis 12. Accordingly, we next sought to determine the feasibility of treating hepatic fibrosis by inhibiting Notch signaling in macrophages.

Transcription factors are important targets for the treatment of diseases caused by overactivated signaling pathways. TFD ODNs are short, double-stranded DNA molecules representing a novel class of nucleic acid-based drugs 17. TFD ODNs can contain the consensus DNA binding sequence for a specific transcription factor normally found in the promoter regions of its target genes. SiRNAs or shRNAs are widely applied for the downregulation of the expression of transcription factors by promoting the degradation of their transcripts. However, transcription factors that have already been produced retain their activities until they are degraded. In contrast, TFD ODNs can downregulate the expression of target genes faster than RNAi by acting as decoys for the binding of existing transcription factors 17,18. Nevertheless, TFD ODNs also have their limitations 18. First, they are prone to degradation by nucleases and are unstable. Second, the negative charge of the nucleotides hampers their cellular uptake. Third, the systemic administration of TFD ODNs is likely to result in more severe adverse effects, particularly if the target transcription factor has important, multifunctional roles. Hence, the structure of TFD ODNs must be modified to enhance their stability and cellular uptake. This includes endowing single-stranded ODNs with the ability to form hairpins and circular ODNs to assume a dumbbell configuration, as well as introducing locked nucleic acids or internucleotidic phosphorothioate bonds 17. Furthermore, the application of tissue- or cell-specific delivery systems, such as nanovectors, can reduce the side effects associated with TFD ODNs.

Exosomes are nano-sized phospholipid bilayer-enclosed vesicles secreted by living cells and are thought to have enormous potential as natural drug delivery vehicles 13,32. Drugs can be loaded into exosomes by electroporation, co-incubation, ultrasound treatment, saponin treatment, density gradient ultracentrifugation, and freeze-thaw extrusion 32. Owing to their endogenous nature, exosomes have lower immunogenicity compared with exogenous nanocarriers 33. Interestingly, in the liver, the major site for exosome removal from the systemic circulation, exosomes are predominantly taken up by macrophages 15,16. This indicates that exosomes may represent a natural delivery system for the targeting of hepatic macrophages.

In this study, we described a hairpin decoy ODN for RBP-J containing three phosphorothioate-modified sites at each end to enhance its stability. We found that RBP-J decoy ODNs could inhibit Notch signaling activation and could be loaded into HEK293T-derived exosomes by electroporation. We further observed that exosomes administered *via* tail vein injection were mainly taken up by hepatic macrophages in mice with hepatic fibrosis, and that RBP-J decoy ODNs delivered by exosomes could efficiently inhibit Notch signaling in macrophages and ameliorate hepatic fibrosis in these animals. Some issues related to the application of TFD ODNs remain to be solved, such as how to promote their translocation into the nucleus to exert their activity. Linking nuclear localization signals (NLSs) to decoy peptide nucleic acids may be one solution. Surface receptors in exosomes can also be modified to target liver sinusoidal endothelial cells (LSECs) and deliver Notch inhibitors with the aim of treating hepatic fibrosis. Combined, our findings indicate that the infusion of exosomes loaded with RBP-J decoy ODNs represents a promising therapeutic strategy for the treatment of hepatic fibrosis that merits further investigation.

## Supplementary Material

Supplementary figures and table.

## Figures and Tables

**Figure 7:**
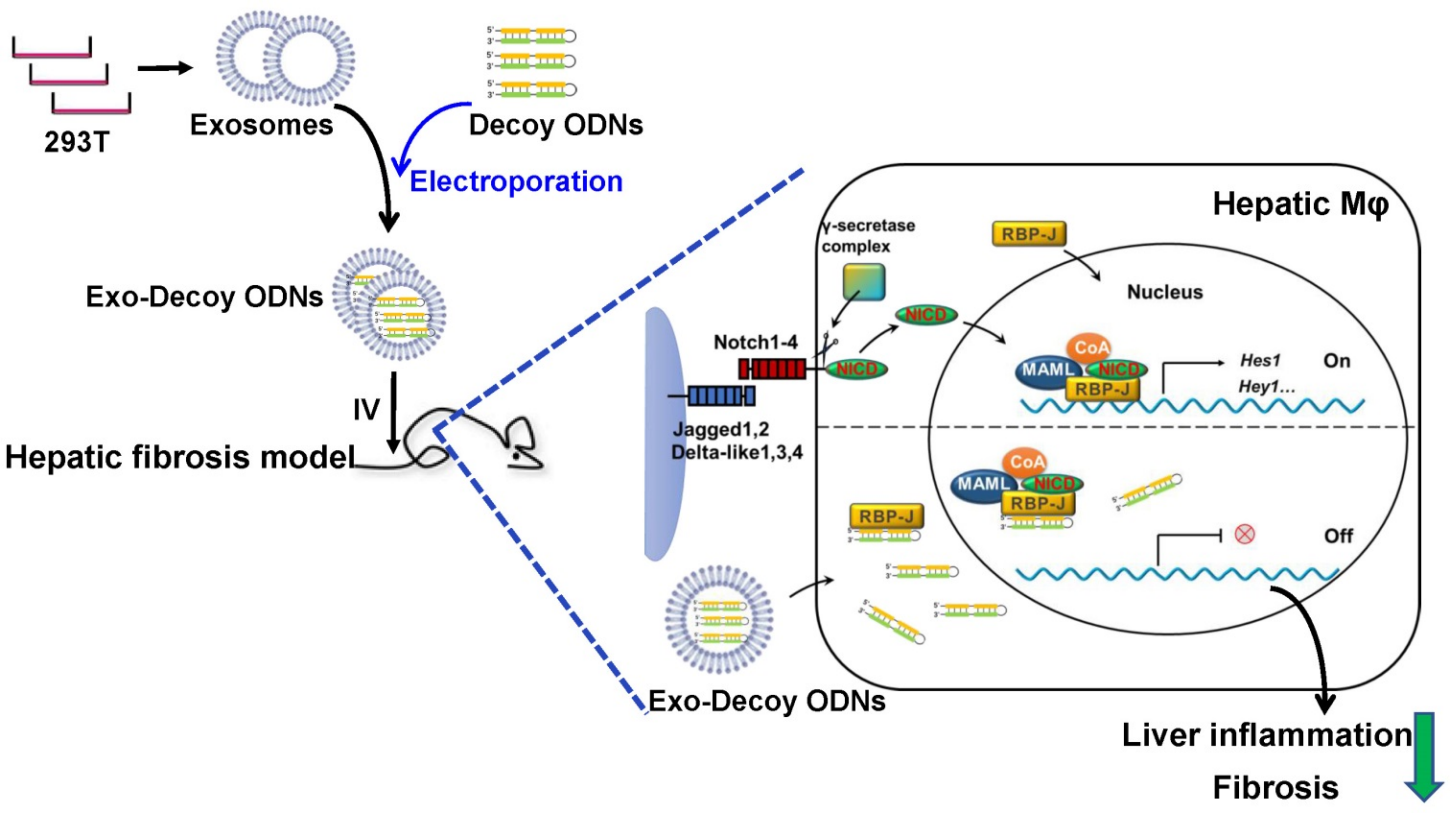
** Schematic summary of this study.** RBP-J decoy oligodeoxynucleotides (ODNs) were loaded into HEK293T-derived exosomes by electroporation. Next, exosomes loaded with RBP-J decoy ODNs (exosomes-RBP-J decoy ODNs) were injected into mice *via* the tail vein. The infused exosomes-RBP-J decoy ODNs were mainly taken up by hepatic macrophages, resulting in the inhibition of Notch signaling activation in these cells and, finally, the amelioration of liver inflammation and fibrosis.

## References

[1] Mokdad AA, Lopez AD, Shahraz S, Lozano R, Mokdad AH, Stanaway J (2014). Liver cirrhosis mortality in 187 countries between 1980 and 2010: a systematic analysis. BMC Med.

[2] Kisseleva T, Brenner D (2021). Molecular and cellular mechanisms of liver fibrosis and its regression. Nat Rev Gastroenterol Hepatol.

[3] Ramachandran P, Iredale JP, Fallowfield JA (2015). Resolution of liver fibrosis: basic mechanisms and clinical relevance. Semin Liver Dis.

[4] Alison MR, Lin WR (2018). Macrophages come on tap for liver fibrosis therapy. Hepatology.

[5] Wen Y, Lambrecht J, Ju C, Tacke F (2021). Hepatic macrophages in liver homeostasis and diseases-diversity, plasticity and therapeutic opportunities. Cell Mol Immunol.

[6] van der Heide D, Weiskirchen R, Bansal R (2019). Therapeutic Targeting of Hepatic Macrophages for the Treatment of Liver Diseases. Front Immunol.

[7] Duffield JS, Forbes SJ, Constandinou CM, Clay S, Partolina M, Vuthoori S (2005). Selective depletion of macrophages reveals distinct, opposing roles during liver injury and repair. J Clin Invest.

[8] Artavanis-Tsakonas S, Rand MD, Lake RJ (1999). Notch signaling: cell fate control and signal integration in development. Science.

[9] Wang YC, He F, Feng F, Liu XW, Dong GY, Qin HY (2010). Notch signaling determines the M1 versus M2 polarization of macrophages in antitumor immune responses. Cancer Res.

[10] Zhang W, Xu W, Xiong S (2010). Blockade of Notch1 signaling alleviates murine lupus via blunting macrophage activation and M2b polarization. J Immunol.

[11] Xu H, Zhu J, Smith S, Foldi J, Zhao B, Chung AY (2012). Notch-RBP-J signaling regulates the transcription factor IRF8 to promote inflammatory macrophage polarization. Nat Immunol.

[12] He F, Guo FC, Li Z, Yu HC, Ma PF, Zhao JL (2015). Myeloid-specific disruption of recombination signal binding protein Jĸ ameliorates hepatic fibrosis by attenuating inflammation through cylindromatosis in mice. Hepatology.

[13] EL Andaloussi S, Mäger I, Breakefield XO, Wood MJ (2013). Extracellular vesicles: biology and emerging therapeutic opportunities. Nat Rev Drug Discov.

[14] Valadi H, Ekström K, Bossios A, Sjöstrand M, Lee JJ, Lötvall JO (2007). Exosome-mediated transfer of mRNAs and microRNAs is a novel mechanism of genetic exchange between cells. Nat Cell Biol.

[15] Imai T, Takahashi Y, Nishikawa M, Kato K, Morishita M, Yamashita T (2015). Macrophage-dependent clearance of systemically administered B16BL6-derived exosomes from the blood circulation in mice. J Extracell Vesicles.

[16] Zhang G, Huang X, Xiu H, Sun Y, Chen J, Cheng G (2020). Extracellular vesicles: Natural liver-accumulating drug delivery vehicles for the treatment of liver diseases. J Extracell Vesicles.

[17] Hecker M, Wagner AH (2017). Transcription factor decoy technology: A therapeutic update. Biochem Pharmacol.

[18] Farahmand L, Darvishi B, Majidzadeh-A K (2017). Suppression of chronic inflammation with engineered nanomaterials delivering nuclear factor kappaB transcription factor decoy oligodeoxynucleotides. Drug Deliv.

[19] Mehta M, Paudel KR, Shukla SD, Allam VSRR, Kannaujiya VK, Panth N (2021). Recent trends of NFkappaB decoy oligodeoxynucleotide-based nanotherapeutics in lung diseases. J Control Release.

[20] Tun T, Hamaguchi Y, Matsunami N, Furukawa T, Honjo T, Kawaichi M (1994). Recognition sequence of a highly conserved DNA binding protein RBP-J kappa. Nucleic Acids Res.

[21] Liu W, Hou Y, Chen H, Wei H, Lin W, Li J (2011). Sample preparation method for isolation of single-cell types from mouse liver for proteomic studies. Proteomics.

[22] Yue KY, Zhang PR, Zheng MH, Cao XL, Cao Y, Zhang YZ (2019). Neurons can upregulate Cav-1 to increase intake of endothelial cells-derived extracellular vesicles that attenuate apoptosis via miR-1290. Cell Death Dis.

[23] Alvarez-Erviti L, Seow Y, Yin H, Betts C, Lakhal S, Wood MJ (2011). Delivery of siRNA to the mouse brain by systemic injection of targeted exosomes. Nat Biotechnol.

[24] Qin H, Wang J, Liang Y, Taniguchi Y, Tanigaki K, Han H (2004). RING1 inhibits transactivation of RBP-J by Notch through interaction with LIM protein KyoT2. Nucleic Acids Res.

[25] Jeppesen DK, Fenix AM, Franklin JL, Higginbotham JN, Zhang Q, Zimmerman LJ (2019). Reassessment of exosome composition. Cell.

[26] Adams JM, Jafar-Nejad H (2019). The roles of Notch signaling in liver development and disease. Biomolecules.

[27] Zhu C, Kim K, Wang X, Bartolome A, Salomao M, Dongiovanni P (2018). Hepatocyte Notch activation induces liver fibrosis in nonalcoholic steatohepatitis. Sci Transl Med.

[28] Yue Z, Jiang Z, Ruan B, Duan J, Song P, Liu J (2021). Disruption of myofibroblastic Notch signaling attenuates liver fibrosis by modulating fibrosis progression and regression. Int J Biol Sci.

[29] Duan JL, Ruan B, Yan XC, Liang L, Song P, Yang ZY (2018). Endothelial Notch activation reshapes the angiocrine of sinusoidal endothelia to aggravate liver fibrosis and blunt regeneration in mice. Hepatology.

[30] van Es JH, van Gijn ME, Riccio O, van den Born M, Vooijs M, Begthel H (2005). Notch/gamma-secretase inhibition turns proliferative cells in intestinal crypts and adenomas into goblet cells. Nature.

[31] Richter LR, Wan Q, Wen D, Zhang Y, Yu J, Kang JK (2020). Targeted delivery of Notch inhibitor attenuates obesity-induced glucose intolerance and liver fibrosis. ACS Nano.

[32] Li M, Li S, Du C, Zhang Y, Li Y, Chu L (2020). Exosomes from different cells: Characteristics, modifications, and therapeutic applications. Eur J Med Chem.

[33] Liao W, Du Y, Zhang C, Pan F, Yao Y, Zhang T (2019). Exosomes: The next generation of endogenous nanomaterials for advanced drug delivery and therapy. Acta Biomater.

