# Supplementary material for: Exosome-mediated delivery of RBP-J decoy oligodeoxynucleotides ameliorates hepatic fibrosis in mice
Source: Theranostics. 2022 Jan 24;12(4):1816–28. doi: 10.7150/thno.69885 (PMC8825583; doi:10.7150/thno.69885)
Supplement: Supplementary file 1 — Supplementary figures and table. [file thnov12p1816s1.pdf]

Supporting Materials

Exosome-mediated delivery of RBP-J decoy oligodeoxynucleotides ameliorates hepatic fibrosis in mice

Fei He[1,*] (hefei_hefei@163.com)

Wei-Na Li[2,*] (liweina228@163.com)

Xin-Xin Li[3,*] (lixinxin1989123@nwpu.edu.cn)

Kang-Yi Yue[4] (yuekangyi@qq.com)

Juan-Li Duan[1] (duan_juan_li@126.com)

Bai Ruan[1,4] (bairuan@fmmu.edu.cn)

Jing-Jing Liu[1] (ljj05080086@126.com)

Ping Song[1] (songping000123@163.com)

Zhen-Sheng Yue[1] (18392181767@163.com)

Kai-Shan Tao[1,#] (taokaishan0686@163.com)

Lin Wang[1,#] (fierywang@163.com)

[1] Department of Hepatobiliary Surgery, Xi-Jing Hospital, Fourth Military Medical University, Xi'an 710032, China.

[2] School of Basic Medicine, Fourth Military Medical University, Xi'an 710032, China.

[3] Xi'an Key Laboratory of Stem Cell and Regenerative Medicine, Institute of Medical Research, Northwestern Polytechnical University, Xi'an 710072, China.

[4] State Key Laboratory of Cancer Biology, Department of Biochemistry and Molecular Biology, Fourth Military Medical University, Xi'an 710032, China.

* These authors contributed equally to this study.

# Corresponding authors.

Correspondence to: Kai-Shan Tao, Department of Hepatobiliary Surgery, Xi-Jing Hospital, Fourth Military Medical University, Xi'an 710032, China, Email: taokaishan0686@163.com; Fax: (86)-29-84771273; Lin Wang, Department of Hepatobiliary Surgery, Xi-Jing Hospital, Fourth Military Medical University, Xi'an 710032, China, Email: fierywang@163.com; Fax: (86)-29-83246270

Supplementary Methods

ChIP

ChIP assays were performed with SimpleChIP Enzymatic Chromatin IP Kit (CST), according to the manufacturer's instructions. Briefly, HeLa cells were co-transfected with pCMV-RBPJ-Flag and Decoy RBP-J, or Decoy Ctrl ODNs for 24-36 h. The cells were then cross-linked with 1% formaldehyde for 10 min, and quenched with 125 mM glycine for 5 min at room temperature. Chromatin was digested by Micrococcal Nuclease, and sheared by sonication. The length of chromatin was around 150-900 bp. The fragmented chromatin was subjected to immunoprecipitation with anti-Flag antibody (CST), with anti-Histone H3 antibody as a positive control, or with anti-IgG as negative control at 4°C overnight. Protein G magnetic beads were then added to the

mixture and incubated at 4°C for 2 h. DNA was eluted and purified according to the protocol. Human HEY1 promoter fragments (-180~-65) encompassing one RBP-J binding site in input and precipitated DNA were detected by qRT-PCR. ChIP primers are listed in Supporting Table S1.

Hepatic macrophages isolation

Hepatic macrophages were isolated as described (reference: *Proteomics* 2011; 11:3556-3564). In brief, anesthetized mice were perfused with D-Hank's buffer from inferior vena cava, followed by Hank's buffer containing collagenase IV (0.2g/L) (Sigma). Liver NPCs was prepared and hepatocytes were eliminated by three centrifugations at 50 g for 3 min. The cell fraction were collected by using successive gradient centrifugations on 8.2% and 17.6% Iodixanol (Optiprep^{TM}, Axis-Shield, Oslo, Norway), incubated with phycoerythrin (PE)-conjugated anti-F4/80 antibody (eBioscience, San Diego, CA, USA) and then with anti-PE microbeads (Miltenyi Biotec, Bergisch Gladbach, Germany). Then, hepatic macrophages were purified by MACS according the recommended protocol.

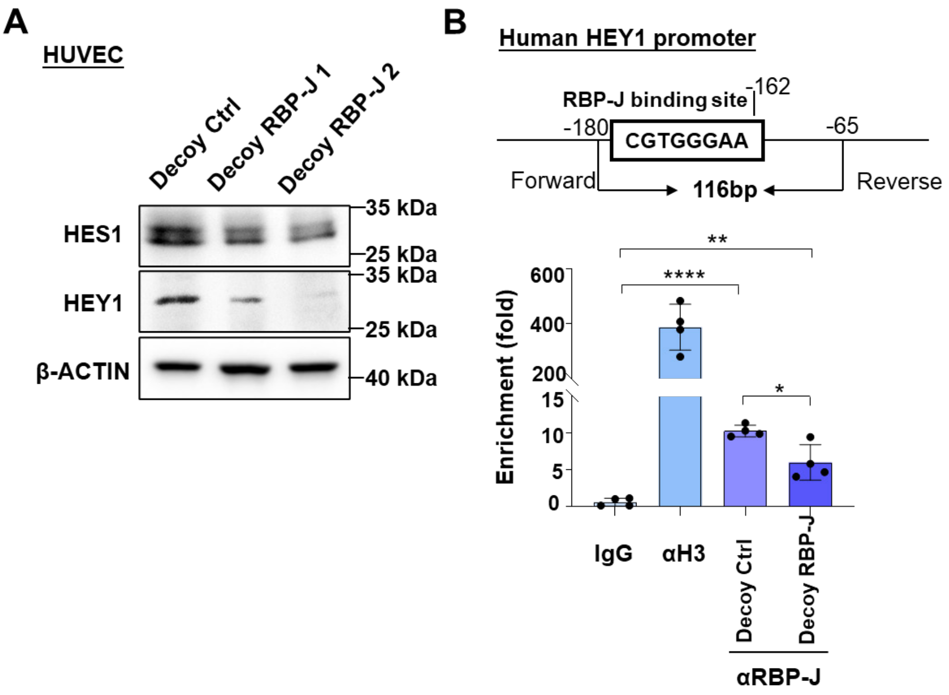

Figure S1. RBP-J decoy ODNs impaired the activation of Notch signaling in HUVEC and HeLa cells.

(A) The expression of HES1 or HEY1 was determined by Western blot in HUVEC.

(B) ChIP assay. HeLa cells were co-transfected with pCMV-RBPJ-Flag and RBP-J decoy, or control ODNs. ChIP was performed with anti-Flag, rabbit IgG or anti-Histone H3, and protein G magnetic beads. Human HEY1 promoter fragments (-180~-65) encompassing one RBP-J binding site in input and precipitated DNA were detected by qRT-PCR.

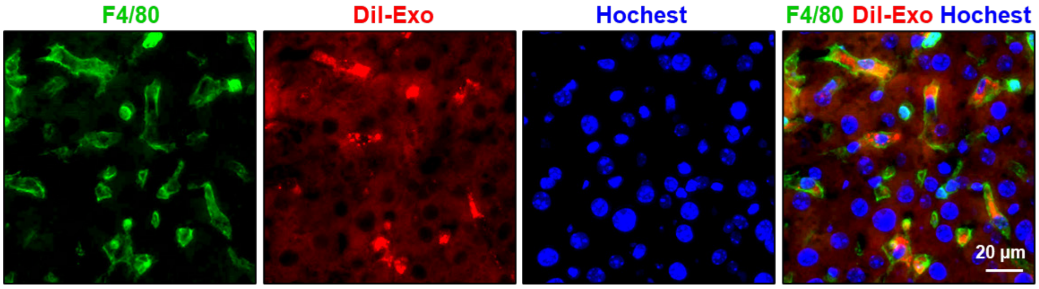

Figure S2. Tail vein-injected exosomes were mainly taken up by hepatic macrophages in BDL-induced mice. Liver sections were stained with anti-mouse F4/80 antibody and analyzed by fluorescence microscope 6 h after the DiI-exosomes injection. Nuclei were counterstained using Hoechst 33258.

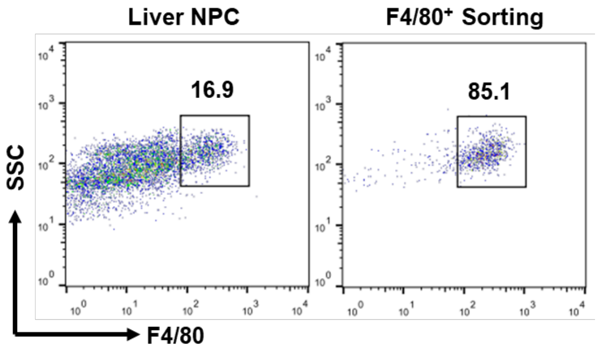

Figure S3. The liver nonparenchymal cells (NPCs) and sorted F4/80$^+$ cells were analyzed by FACS. Hepatic macrophages were isolated using magnetic bead cell sorting (MACS).

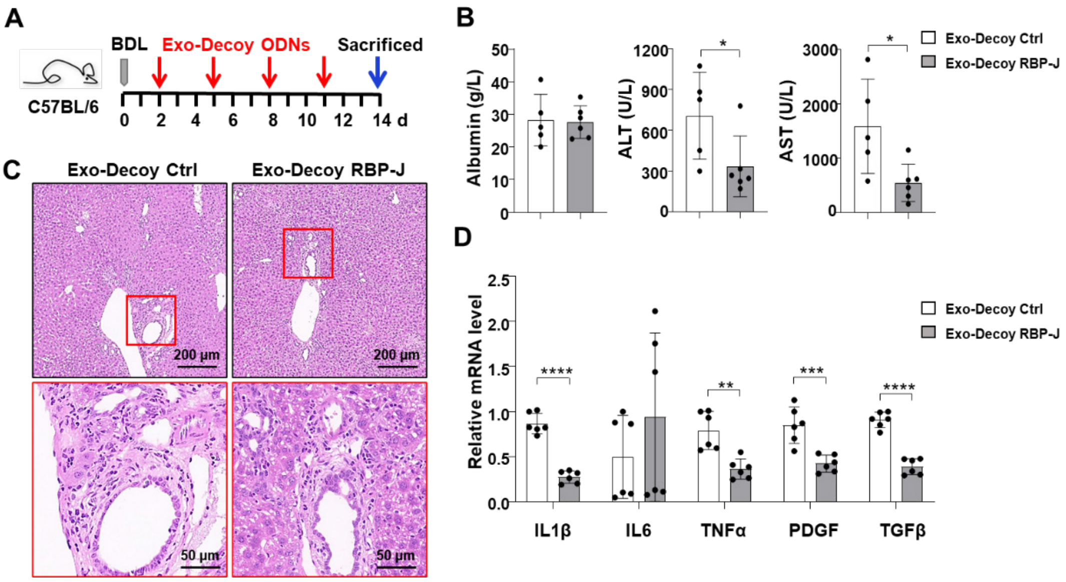

Figure S4. Exosomes loading RBP-J decoy ODNs attenuated liver inflammation after the induction of liver fibrosis by bile duct ligation (BDL).

(A) Schematic representation of the experimental procedure. Hepatic fibrosis was induced by extrahepatic cholestasis resulted from BDL. Exosomes loading RBP-J decoy, or control ODNs (exosomes/Decoy ODNs = 200 ng/2.5 nmol) were infused into mice four times by tail vein injection. Mice were analyzed 2 weeks after BDL operation. (B) Liver sections were stained by H&E staining and the lower row of photomicrographs were a higher magnification of the red frames in the upper row. (C) Serum albumin, AST and ALT levels of the mice were measured. (D) The mRNA levels of IL1β, IL6, TNF-α, PDGF-B and TGFβ in the livers were determined by qRT-PCR. Bars = means ± SD, *, $P < 0.05$, **, $P < 0.01$, ***, $P < 0.001$.

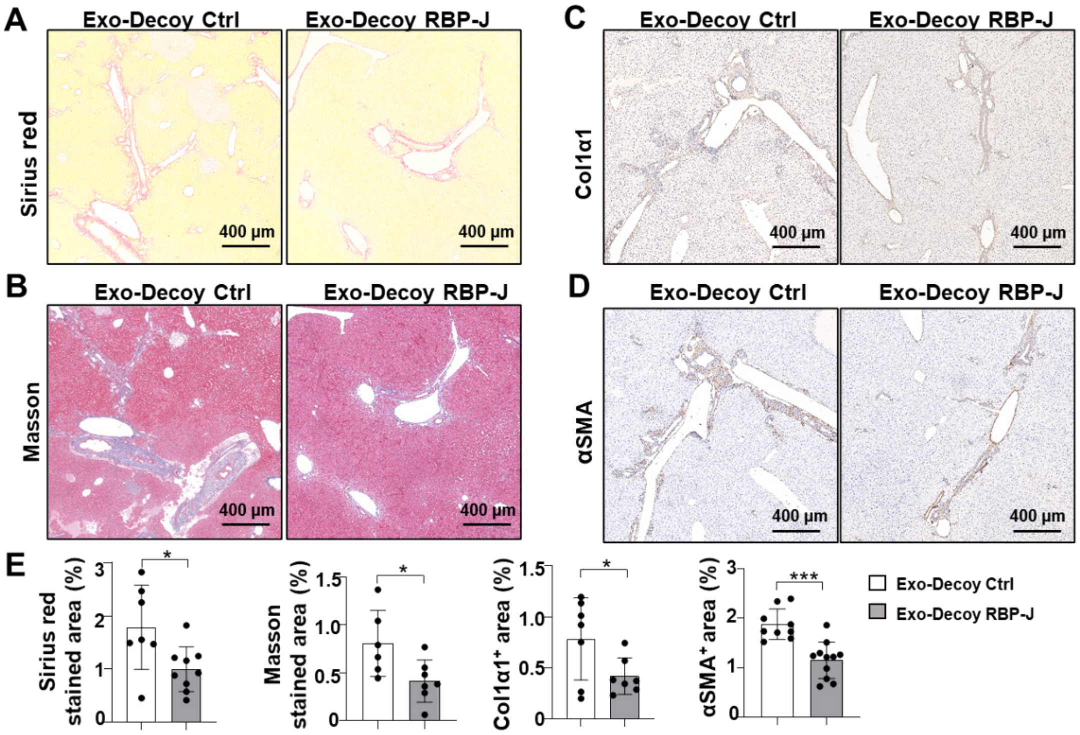

Figure S5. Exosomes loading RBP-J decoy ODNs ameliorated BDL-induced liver fibrosis.

(A) Liver sections were stained with Sirius red staining. (B) Liver sections were stained with Masson staining. (C) Liver sections were subjected to immunohistochemical staining for Col1α1. (D) Liver sections were stained with anti-αSMA. (E) The positive areas of Sirius red staining in (A), Masson staining in (B), Col1α1 in (C) and αSMA in (D) were quantitatively compared. Data were analyzed using unpaired t test. Bars = means ± SD, *, $P < 0.05$, ***, $P < 0.001$.

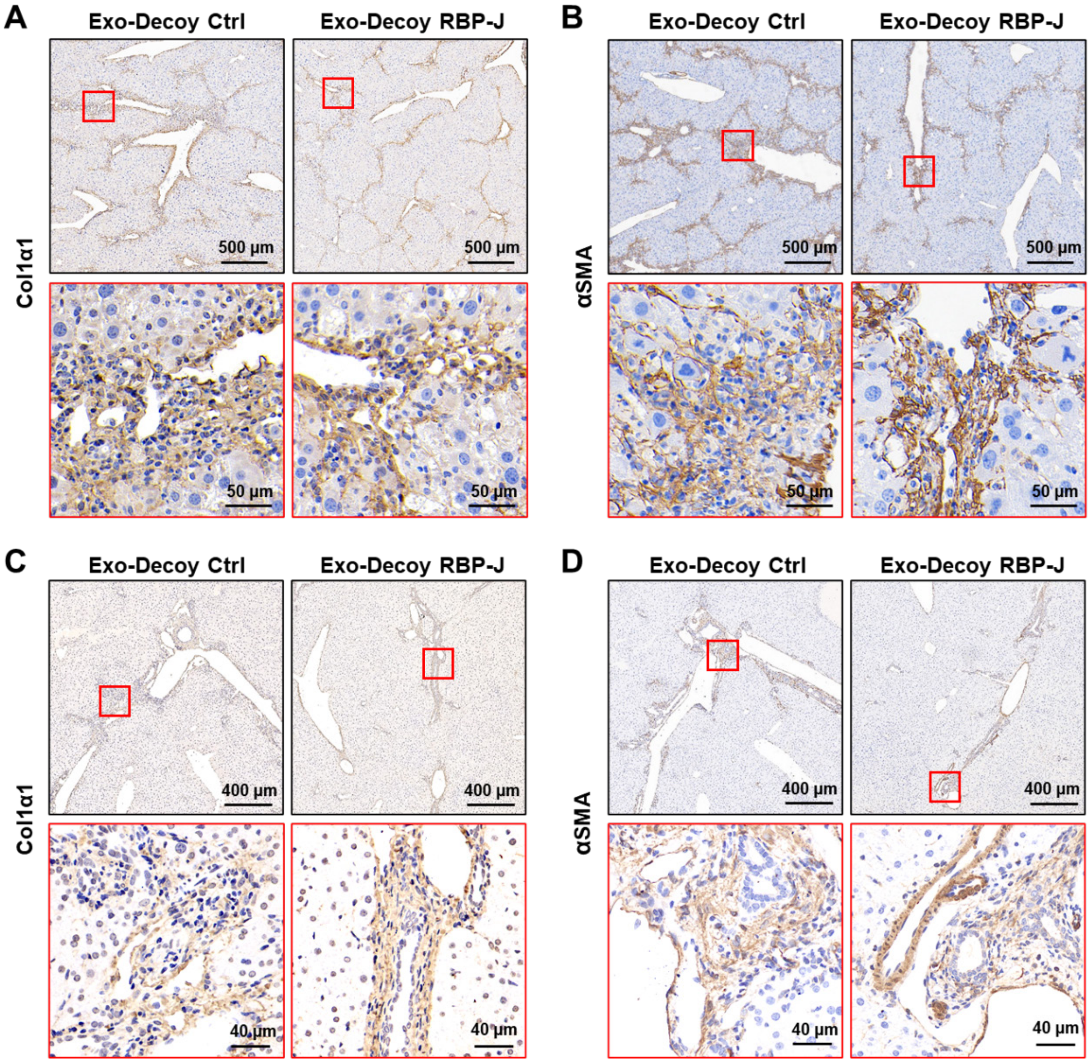

Figure S6. Liver sections were subjected to immunohistochemical staining for Col1α1 and αSMA, and a higher magnification was shown.

(A) and (B) were CCl_4-induced hepatic fibrosis, (C) and (D) were BDL-induced hepatic fibrosis. (A) and (C) were stained for anti-Col1α1. (B) and (D) were stained for anti-αSMA. The lower row of photomicrographs were a higher magnification of the red frames in the upper row.

Table S1. Sequences of primers used in the study

Gene	Forward/Reverse	Sequence
β-actin	Forward	5’-CATCCGTAAAGACCTCTATGCCAAC-3’
	Reverse	5’-ATGGAGCCACCGATCCACA-3’
IL-1β	Forward	5’-TCCAGGATGAGGACATGAGCAC-3'
	Reverse	5’-GAACGTCACACACCAGCAGGTTA-3’
IL-6	Forward	5’-CCACTTCACAAGTCGGAGGCTTA-3’
	Reverse	5’-GCAAGTGCATCATCGTTGTTCATAC-3’
TNF-α	Forward	5’-CAGGAGGGAGAACAGAAACTCCA-3’
	Reverse	5’-CCTGGTTGGCTGCTTGCTT-3’
iNOS	Forward	5’-GCAGAGATTGGAGGCCTTGTG -3’
	Reverse	5’-GGGTTGTTGCTGAACTTCCAGTC-3’
TGFβ	Forward	5’-GACCGCAACAACGCCATCTA-3’
	Reverse	5’-GGCGTATCAGTGGGGGTCAG-3’
PDGF-B	Forward	5’-TACCTGCGTCTGGTCAGC-3’
	Reverse	5’-GCTCGGGTCATGTTCAAG-3’
Cyld	Forward	5’-ACCCTACTGGGAAGAACGGAT-3’
	Reverse	5’-CGGTCTTGGATGTACTGTCCTAT-3’
Hes1	Forward	5’-AAAGACGGCCTCTGAGCAC-3’
	Reverse	5’-GGTGCTTCACAGTCATTTCCA-3’
HES1 (human)	Forward	5’-TGGAAATGACAGTGAAGCACCTC-3’
	Reverse	5’-TCGTTCATGCACTCGCTGAAG-3’
HEY1 (human)	Forward	5’-AGCAAAGCGTTGACAAATCAGATG-3’
	Reverse	5’-CAGCAGCTGGTCAGATGGATTC-3’
HEY2 (human)	Forward	5’-AAGGCGTCGGGATCGGATAA-3’
	Reverse	5’-AGAGCGTGTGCGTCAAAGTAG-3’
GAPDH (human)	Forward	5’-TGCACCACCAACTGCTTAGC-3’
	Reverse	5’-GGCATGGACTGTGGTCATGAG-3’
HEY1 (ChIP)	Forward	5’-AAGGGGCTCAGCGTGGGAAA-3’
	Reverse	5’-AGTTAACTACAGCGGCGCCTCTCC-3’
MiR-140-5p	Forward	5’-GCCAGTGGTTTTACCCTATGGTAG-3’
U6	Forward	5’-GGATGACACGCAAATTCGTGAAGC-3’
